# Biphasic Calcium Phosphate Ceramics for Bone Regeneration and Tissue Engineering Applications

**DOI:** 10.3390/ma3020815

**Published:** 2010-01-29

**Authors:** Sonja Ellen Lobo, Treena Livingston Arinzeh

**Affiliations:** 1 Department of Morphology, Federal University of São Paulo, Rua Botucatu, 740, CEP 04023-900, São Paulo, SP, Brazil; E-Mail: sonjael@terra.com.br; 2 Department of Biomedical Engineering, New Jersey Institute of Technology, University Heights; 614 Fenster Hall, Newark, NJ 07102-1982, USA

**Keywords:** calcium phosphate ceramics, bone reconstruction, tissue engineering, stem cell

## Abstract

Biphasic calcium phosphates (BCP) have been sought after as biomaterials for the reconstruction of bone defects in maxillofacial, dental and orthopaedic applications. They have demonstrated proven biocompatibility, osteoconductivity, safety and predictability in *in vitro*, *in vivo* and clinical models. More recently, *in vitro* and *in vivo* studies have shown that BCP can be osteoinductive. In the field of tissue engineering, they represent promising scaffolds capable of carrying and modulating the behavior of stem cells. This review article will highlight the latest advancements in the use of BCP and the characteristics that create a unique microenvironment that favors bone regeneration.

## 1. Introduction

Calcium phosphate ceramics have been widely applied as bone substitutes, coatings, cements, drug delivery systems and tissue engineering scaffolds due to their resemblance to the mineral portion of the bone tissue, relative ease in processing and good cell attachment [1,2,3,4]. Its biocompatibility, safety, predictability, unlimited availability, lower morbidity for the patient and cost effectiveness represent important advantages over autografts and allografts [3,5] and make them a good choice for reconstructive surgery, orthopaedics, dentistry, maxillo and craniofacial surgeries, spinal arthrodesis and neurosurgery [3,5,6,7,8,9,10,11,12,13].

Over the years, several modifications on parameters such as sintering temperature, pH and purity of the starting products have given rise to calcium phosphates with distinct chemical and physical characteristics such as specific surface areas, surface energy, surface charge, roughness and porosity [1,3,11,14,15]. Macropores (diameter > 100 μm) and micropores (diameter < 10 μm) can be created in bioceramics with the use of porogens/pore-formers and heat treatment [14]. As a consequence, it is possible to have a biomaterial that improves the adhesion, proliferation and differentiation of cells, which leads to better osteoconductivity, bioactivity and mechanical properties with less brittleness [11,16,17]. Although studies demonstrate that some bioceramics have osteoinductive properties, the cellular and molecular mechanisms that explain such a process are not completely understood. A few theories have considered the physicochemical and structural characteristics of the bioceramic. It has been described that a high specific surface area, which can be achieved by increasing the number of micropores, is essential for osteoinduction [15,16,18,19,20,21]. The presence of concavities, which are present at the walls of macropores, has also been considered a key point, for they resemble the geometric-dependent event of bone formation [22,23]. Furthermore, the dissolution of the surface causes a supersaturation of calcium and phosphate ions, which leads to their reprecipitation and the formation of a biological apatite layer [14,16,18,24,25,26]. This property allows bone-bonding with the bioceramic and influences its osteoinduction potential.

Among the calcium phosphate ceramics, the biphasic calcium phosphates (BCP), which are composed of different concentrations of the stable phase, hydroxyapatite (HA), and the more soluble phase, usually composed of β-tricalcium phosphate (β-TCP), have presented significant advantages over other calcium phosphate ceramics due to their controlled bioactivity and balance between resorption/solubilization which guarantees the stability of the biomaterial while promoting bone ingrowth [27,28]. Depending upon the concentration of the more stable and soluble phases, it is possible to obtain a ceramic that can be applied to large bone defects, in load bearing areas, and as customized pieces which will maintain their shape over long periods of time [8,28,29].

The nature, timing and progression of bone formation is dependent upon the chemistry and physical properties of the bioceramic [1,11]. This review paper describes the physicochemical characteristics of BCP that create a unique microenvironment for bone formation and their use as a promising tissue engineering scaffold for bone regeneration.

## 2. Physicochemical Properties of BCP that Influence Bone Formation

The type of biological response by the host is critical for bone formation on a bioceramic surface and this response is dependent upon the ceramic’s chemical composition and physical structure. A bioceramic can be classified according to the type of interface formed between the bioceramic and the host tissue. A bioceramic can be classified as inert (where there is a minor fibrous reaction by the host on the surface of the biomaterial) and bioactive (where there is a direct biochemical and biological bond at the interface with the adjacent bone tissue, via the formation of an apatite layer at the surface of the biomaterial). This interface influences the rate and type of bone formation and the stability and mechanical strength at the interface, which can play a role in the success or failure of the implant [12,30].

**Figure 1:**
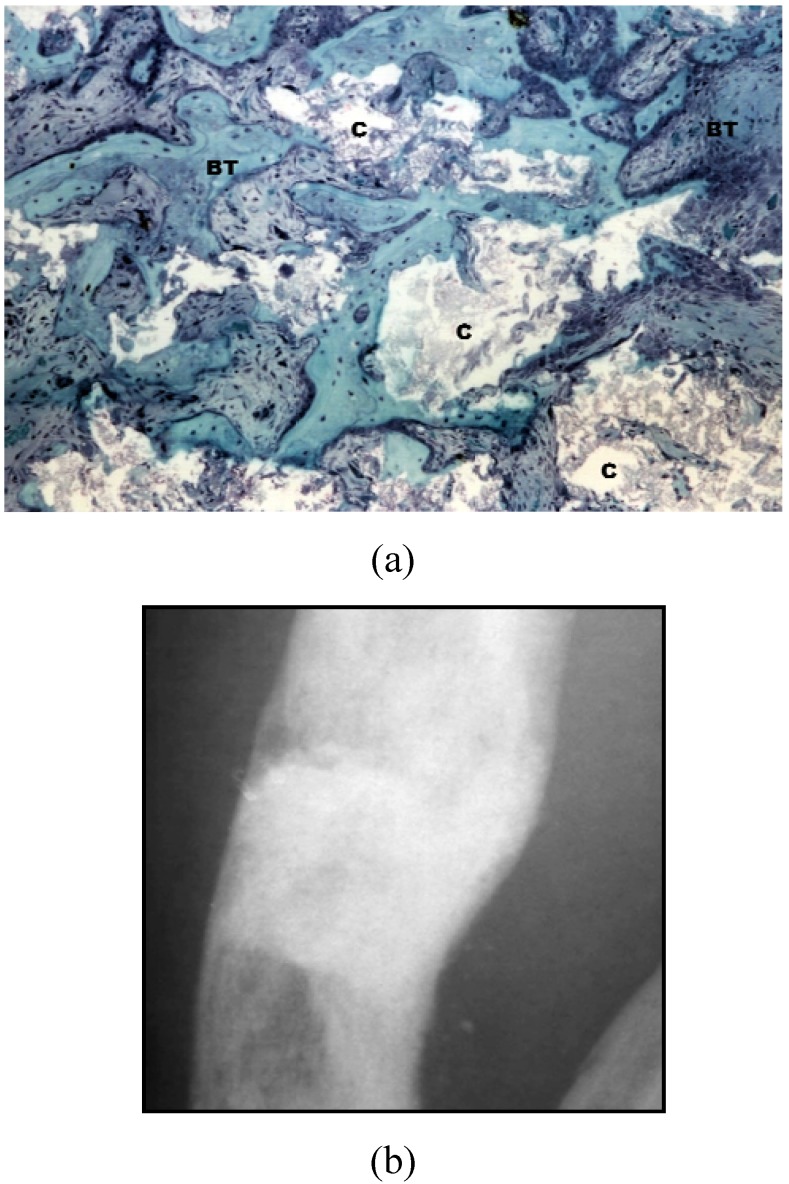
Histological and radiographical images of a BCP (Osteosynt ®) in granular form (40–60 mesh or approx. 250 to 420 μm). (a) Histological image after implantation in a rabbit femur (250X, Toluidine blue); (b) Radiographic image after the reconstruction of a tibial defect in a human (surgical procedure performed by Dr C. A. Garrido). BT = bone tissue; C = ceramics.

Thus, three generations of bioceramics have been described: the first is characterized as inert materials (e.g., alumina and zirconia) in which the bone substitutes cause a mild inflammatory response and result in a minor fibrous encapsulation [31]. The second generation corresponds to bioactive biomaterials which are explained by a sequential transition between the nucleation of an amorphous calcium phosphate layer, formation of octacalcium phosphate and finally, the maturation into a calcium deficient carbonated hydroxyapatite layer [31]. Bioactive glasses, highly porous HA and macroporous BCP ceramics are examples of biomaterials with such a property [14,32]. Finally, the third generation corresponds to bioactive materials with different porosities, better mechanical properties and chemical compositions that allow improved ion exchange and leads to osteogenesis [12,31,32]. BCP ceramics can be fabricated that promote the formation of bone with mature bone structure, including a Haversian-like system, and a low level of an inflammatory response [17,26,31,32] (Figure 1).

Bone formation within a ceramic is a multifactor process that is regulated by several aspects such as chemical composition, resorption and dissolution rates, physical structure (e.g., geometry of the pores, porosity as well as surface topography) and implantation site. The chemical composition of a bioceramic influences the rate of solubilization/resorption as well as its bioactivity. For instance, while HA is slowly resorbed and/or solubilized, calcium sulphate, α-TCP and β-TCP undergoes a much faster process of resorption. In addition, the rate of resorption can be manipulated by ionic substitutions of some salts. The two most described examples are the carbonate and the silicate-substitute calcium phosphates (Si-CaP) that can present an increased rate of resorption when compared to the stoichiometric HA [11,33,34]. A completely resorbable ceramic has been the goal of several studies; however, a high rate of resorption or solubilization can interfere with bone formation as the biomaterial may degrade faster than the rate of bone formation. This phenomena leads to a change in the bioceramic’s physical structure, *i.e.,* loss of the concavity in the macropore and the mechanical stability of the surface, which will interfere with cell attachment [16,35]. Moreover, the release of high concentrations of calcium to the microenvironment results in a change of the pH, promotes a mild inflammatory response and favors fibrous tissue formation [11,36]. Furthermore, higher calcium ion levels have been shown to effect osteoclastic activity, varying from its inhibition to its stimulation or no effects [37,38]. Consequently, a ceramic with a lower resorption rate is stable for enough period of time to allow for the formation of new bone by the host tissue [8]. In addition, the release of controlled levels of calcium ions over time favors the formation of an apatite layer, which is necessary for the bioactivity displayed in HA/β-TCP ceramics [15,25,28]. This bioactivity can be responsible for the ceramic’s osteoconductivity and/or osteoinductivity. In osteoconduction, the biomaterial surface supports the growth of mature osteoblasts and direct apposition of bone onto its surface while in osteoinduction, the biomaterial favors the recruitment of immature or undifferentiated cells and stimulates their differentiation towards the osteoblastic lineage and as a consequence, osteogenesis will be stimulated [11,39].

**Figure 2:**
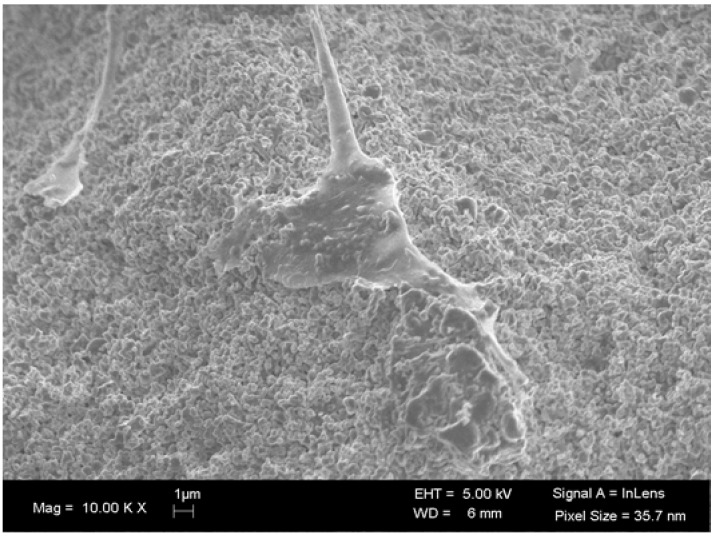
SEM image of human mesenchymal stem cells seeded onto a nanostructured BCP (Osteosynt ®).

The advantages and disadvantages of a bioceramic with high and low resorption or dissolution rates have been widely discussed. The resorption process refers to the cell-mediated mechanism while the dissolution refers to the chemical process that results from the reaction with the surrounding body fluids [1,11,19]. It is important to highlight that nanoparticles (0.1–100 nm) can undergo a process of phagocytosis/endocytosis and not dissolution or resorption [11,40]. Nanoparticles of distinct chemical compositions can enter the cells through gap junctions or hemichannels and can result in DNA damage, alteration of the cell shape and size and cell death [40,41,42,43]. Hydroxyapatite nanoparticles have been studied as a potential therapy for the suppression and apoptosis of osteosarcoma cells, where larger-sized particles appear to be more effective than the smaller ones [43]. The particle size has also been related to the modulation of the inflammatory process: the smaller the particle, the higher the inflammatory process [44] and can also interfere in the *in vitro* differentiation of stem cells [45]. However, this characteristic should not be confused with nanostructured ceramics in which the surface texture improves the attachment and differentiation of osteoprogenitor cells and favors protein adsorption due to its increased surface energy [12,46] (Figure 2).

The physical structure of bioceramics is represented by the surface topography and by the pore structure. Studies have shown that a concave surface favors cell adhesion and proliferation in comparison to a convex surface and is responsible for the beginning of the bone formation process [22,23,47]. The presence of a macroporous structure favors cell ingrowth and blood vessel invasion while the microporosity allows the penetration of body fluids into the implant and increases its bioactivity. Consequently, microporosity can also be a strategy to manipulate the resorption and dissolution rate: the greater the microporosity, the greater the degradation rate [1,11,14,48,49]. Microporosity has been described as one of the factors that influence the intrinsic osteoinductivity of some calcium phosphate ceramics [15,16,18,19,20,21]. Micropores allow for the entrapment and concentration of proteins which, in when contact with undifferentiated cells, will induce their differentiation [47,50].

Therefore, an understanding of the physiochemical characteristics is extremely important when choosing a bioceramic, specifically BCP, as it determines the nature, timing and progress of the tissue formation. In addition, the type and rate of new bone formation depends on the age, systemic condition, metabolism and lifestyle of the patient, the anatomic area that has to be reconstructed, the size of the bone defect, the existence or absence of walls that contain the bioceramic and the surgical technique [8,10,11,13,46]. Different areas of the human skeleton present distinct functional loads, bone density (ratio between the cortical and the medullar zones) and degree of vascularization [46]. This can, in turn, influence the rate of degradation of the ceramic and the overall remodeling process [32].

## 3. BCP Ceramics in Bone Tissue Engineering

A biomaterial that is capable of reconstructing small bone defects may not be suitable to regenerate large bone defects [5], where the current approach includes different types of bone grafts [51]. BCP ceramics have been considered to be a promising scaffold for use with tissue engineering strategies for large bone defect reconstruction. With the aim of improving their osteogenic potential and mechanical properties, such scaffolds have been mixed with autografts, fibrin, platelet concentrate, several growth factors, cytokines and more recently with expanded cells isolated from several tissues [6,9,17,46,52,53,54,55].

**Figure 3:**
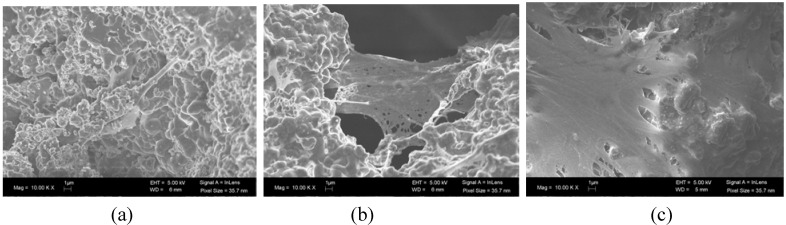
SEM images of the adhesion and proliferation of human mesenchymal stem cells seeded onto a nanostructured BCP ceramic (Osteosynt ®), in the granular form corresponding to 40–60 mesh (approx. 250 to 420 μm). (a) day 1; (b) day 7; (c) day 14.

Autologous and allogeneics stem cells have been isolated from different tissues; however mesenchymal stem cells (MSC) from the bone marrow are the most studied (Figure 3). Whole bone marrow in combination with a scaffold was one of the first strategies described to improve the osteogenic potential of synthetic scaffolds [3]. In subsequent studies, MSC isolated from the bone marrow, which is the osteogenic cell population within the bone marrow, were used in combination with scaffolds to treat bone defects [56]. MSC were expanded in culture and seeded at high densities onto large, porous blocks of BCP. Critically-sized segmental defects in various animal species have been treated with this approach and have formed functional bone tissue *in vivo*. These constructs have been shown to simulate the events of bone formation observed with autologous bone grafts in long bone defects [51].

**Figure 4:**
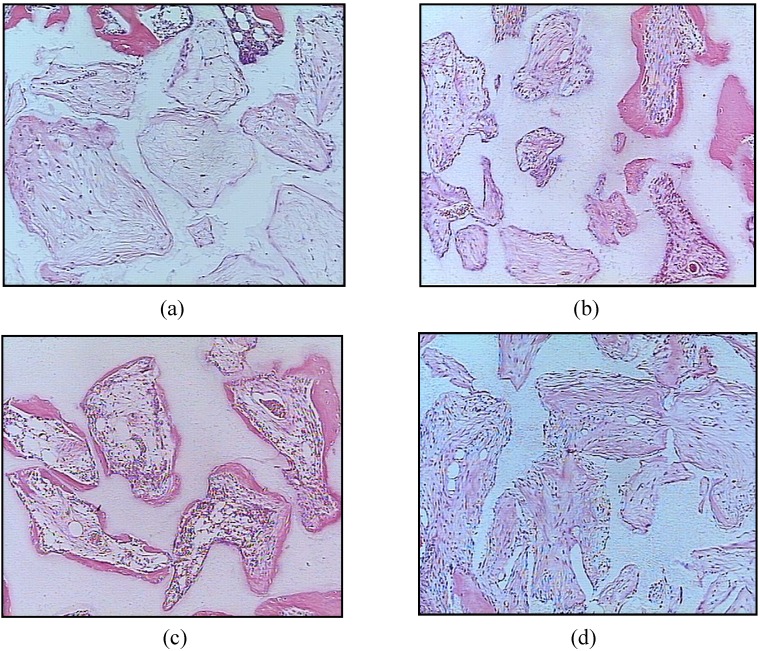
Representative histological micrographs of human MSC loaded onto (a) 100 HA, (b) 63/37 HA/β-TCP, (c) 20/80 HA/β-TCP, and (d) 100 β-TCP implanted for 6 weeks in a subcutaneous mouse model [54]. (Reprinted from Arinzeh, T.L.; Tran, T.; Mcalary, J.; Daculsi, G. A comparative study of biphasic calcium phosphate ceramics for human mesenchymal stem-cell-induced bone formation. *Biomaterials* **2005**, *26*, 3631–3638, with permission from Elsevier.) Bone was detected throughout the porous structure of 20/80 HA/β-TCP and was greater than the other ceramics. (H&E stain, 10× objective). Bone is stained pink, ceramic has a shadowy white appearance, loose connective tissue is stained light pink, cells are stained a dark pink.

Superior bone formation has also been demonstrated in ectopic studies of cells seeded onto calcium phosphate scaffolds in comparison with autograft, allograft or cell-seeded allografts [57]. It has been shown in ectopic sites that the ratio of the HA/β-TCP in the BCP can influence the rate of MSC induced bone formation, where an optimal balance between the more stable and soluble phases must be achieved in order to promote bone tissue formation [54]. 20/80 HA/β-TCP (20 wt % HA: 80 wt % β-TCP) scaffolds seeded with human MSC have been shown to have a higher rate of bone formation over other HA/β-TCP ratios, 100 HA (100% HA) or 100 β-TCP (100% β-TCP) (Figure 4). More recently, MSC have been cultured for a period of time on BCP in order to promote the formation of a bone-like tissue layer on the implant, prior to its implantation [3]. This technique requires, however, more time for preparation, which can be inconvenient for those patients that need a graft immediately [3].

The successful use of stem cells in repairing bone defects may be related to their osteogenic activity and to the paracrine stimulation of other osteoprogenitors cells and vascular ingrowth [51]. The presence of a biomaterial that is able to mimic the three-dimensional characteristics of the bone tissue, to maintain cell viability, proliferation and differentiation is also critical for the success of such a strategy [3,4,52]. Calcium phosphate ceramics are able to maintain the osteogenic capability of stem cells and to induce their differentiation not only *in vitro* but also *in vivo* and it is in agreement with the concept of intrinsic osteoinduction [58,59]. However, the relationship between the cell number/dose and the volume of the scaffold used has to be analyzed for each defect [3,60]. Moreover, the role of stem cells in the defect environment has yet to be defined. It is not clear if the stem cells are important because they synthesize new bone matrix or because they secrete growth factors which can induce bone formation by the host [60]. Consequently, the requirements for the use of a biomaterial, such as a BCP, alone versus in combination with tissue engineering approaches must be defined based on the size and type of the defect.

## 4. Conclusions

BCP ceramics are shown to be biocompatible, bioactive, osteoconductive, safe, predictable and capable of carrying and inducing the differentiation of stem cells. These characteristics associated with the cost, effectiveness, unlimited supply and absence of disease transmission make them a viable alternative to autografts, allografts and others implants.

The ease of tailoring their chemistry, size and shape make them a versatile matrix for the development of strategies to engineer bone formation. BCP ceramics vary according to their chemical composition and physical structures, which in conjunction with the implantation site, form (granules, blocks and customized pieces) and the intrinsic conditions of the patient, can give rise to different rates and patterns of bone formation. The knowledge of such parameters is essential in choosing a BCP for a specific application.
